# Reminiscences of Robert Paul Levine (1927–2022)

**DOI:** 10.1007/s11120-022-00927-6

**Published:** 2022-09-15

**Authors:** Jean-David Rochaix

**Affiliations:** Departments of Molecular Biology and Plant Biology, University of Geneva, 1211 Geneva, Switzerland

**Keywords:** Photosynthesis, Genetics, Chloroplast, *Chlamydomonas reinhardtii*

## Abstract

I present my personal reminiscence of Paul Levine—a highly innovative scientist who did seminal work in photosynthesis. He was among the first to initiate and use a genetic approach toward photosynthesis. He greatly helped in establishing the green unicellular alga *Chlamydomonas reinhardtii* as a powerful model system not only for understanding the function of the photosynthetic apparatus but also for studying its biogenesis and regulation. During the period he spent at Harvard, he made several ground-breaking contributions such as identifying and establishing the order of some components of the photosynthetic electron transport chain as well as determining their genetic origin. He trained many students and post-doctoral fellows several of whom later became prominent in this field and in other areas of plant science.

## Early career

**Fig. 1:**
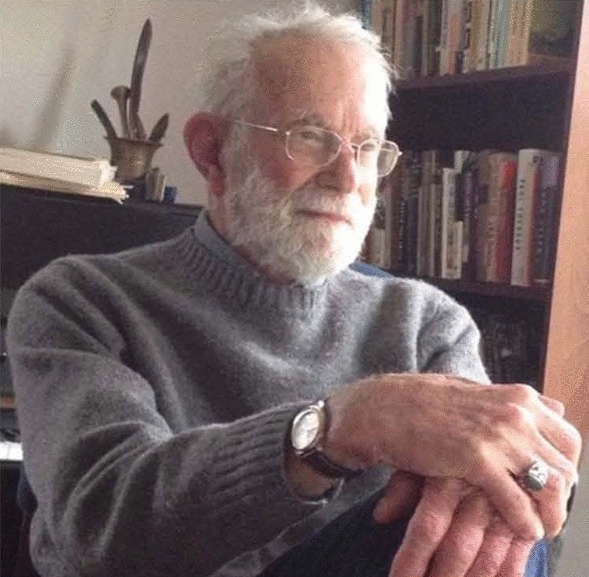
A portrait of Paul Levine in his study at home, provided by his wife Marie-Louise Rouff

Paul Levine passed away on January 25, 2022 at the age of 95; Fig. 1 shows a portrait. He started his research career as assistant professor of biology at Amherst College, MA, USA, in 1951. He joined Harvard University in 1953, and stayed there until 1978 with research focused first on *Drosophila* genetics and later on photosynthesis in *Chlamydomonas*. Subsequently, from1978 to 1992, he worked on the serum complement proteins at the Medical School of Washington University in St. Louis, USA, studying how these proteins bind to cell-surface macromolecules (Law and Levine 2019). He then moved to Stanford’s Hopkins Marine Station, in California, at Pacific Grove from 1992 to 1997 studying bacterial diseases in salmon, the symbiosis between sea anemones and algae as well as biocomposite crystals. He continued to do research as emeritus professor until 2004. For excellent reviews on the research conducted in the laboratory of Paul Levine in photosynthesis and chloroplast biogenesis, see (Goodenough 2015; Togasaki and Surzycki 1998). For an obituary, see https://vineyardgazette.com/obituaries/2022/02/02/robert-paul-levine-95. https://www.chlamycollection.org/celebrating-the-life-of-paul-levine/?utm_source=rss&utm_medium=rss&utm_campaign=celebrating-the-life-of-paul-levine.

## At Harvard

It was during the Harvard period that Paul Levine started an extensive genetic dissection of photosynthesis. He was among the first to use a genetic approach for studying photosynthesis. To do so he wisely chose the green unicellular alga *Chlamydomonas reinhardtii* because of its fast growth, its well-established sexual cycle, and its ability to grow in the absence of photosynthesis on medium containing acetate as reduced carbon source. In this way, he and his collaborators isolated many mutants affected in photosynthesis that were able to grow on acetate-containing medium but not on minimal medium where photosynthetic activity is essential. He also designed a screen in which mutagenized cells were fed ^14^CO_2_ on acetate medium. Cells were then subjected to autoradiography and mutant cells, unable to fix CO_2_, could be identified (Levine 1960). These wide-range screens led to the identification of numerous mutants affected at many steps of photosynthesis including the primary light-driven reactions, photophosphorylation, and the dark reactions of carbon assimilation (Levine 1968). Some mutants obtained through these screens were not primarily affected in photosynthesis, but turned out to be deficient in chloroplast ribosomes revealing a link between photosynthesis and chloroplast biogenesis (Goodenough and Levine 1970; Levine and Goodenough 1970). Bennoun and Levine (1967) designed a screen based on chlorophyll *a* fluorescence emission in which mutant cells could be identified directly on growth plates based on their enhanced chlorophyll *a* fluorescence when photosynthetic electron flow was blocked; this method greatly facilitated the isolation of mutants affected in photosynthesis. With improving technology, this screen was developed to measure fluorescence transients (see e.g., Bennoun and Béal 1998), a powerful method which is still widely used today. Several seminal contributions were made during this period by the Levine laboratory.

First the Z-scheme proposed earlier by Hill and Bendall (1960) (for history of Z-scheme, see Govindjee et al. (2017)) was elegantly confirmed by using mutants blocked at different steps of the photosynthetic electron transport chain. By monitoring the redox state of the different photosynthetic components, their order in the electron transport chain could be determined using mutants affected at specific sites of this chain. As an example, mutants deficient in cytochrome *f* and plastocyanin were identified. Upon illumination of the plastocyanin-minus mutant with PSII light, cytochrome *f* was photo-reduced but could not be photo-oxidized with PSI light (Gorman and Levine 1965). Moreover, photooxidation of an exogenously added reductant, in this case ascorbate and DCIP, by PSI was possible in the cytochrome *f* -deficient mutant but not in the mutant lacking plastocyanin. These experiments unambiguously demonstrated that cytochrome *f* precedes plastocyanin in the electron transport chain, a question which was still unresolved at that time. Another important discovery was the identification of a new component of the electron transport chain located between PSII and cytochrome *f* (Levine 1968). Several years later it was found that the missing component in this mutant is the iron-sulfur Rieske protein which was unknown at that time (Bendall et al. 1986). In addition, this wide photosynthetic mutant hunt identified the first mutants deficient in all major photosynthetic complexes and in photophosphorylation as well as in phosphoribulose kinase in the Calvin–Benson cycle (Levine 1968; Moll and Levine 1970; Sato et al. 1971). These genetic screens led to further surprises. They revealed acetate-requiring mutants that were not primarily affected in photosynthesis. One of them, named *ac*-20, was studied extensively and turned out to be deficient in chloroplast ribosomes thus establishing a link between photosynthesis and chloroplast biogenesis (Levine and Goodenough 1970).

## My association with Paul Levine

It was at that time, in the summer of 1969 that I joined the laboratory of Paul Levine as graduate student at the Biological Laboratories of Harvard. I had just completed my training in Physics in Switzerland and was initially interested by the biophysical part of photosynthesis. Paul gave me a warm welcome and encouraged me to find my own niche in his research group. Some prominent researchers including Nick Gillham and Bob Togasaki had left the lab shortly before my arrival to start their own groups at Duke and Indiana University, respectively (for Togasaki, see Carlson (2022)). Most researchers of the group were studying the photosynthetic electron transport mostly at the biochemical and spectroscopic level and there were some ongoing studies on chloroplast biogenesis.

The existence of chloroplast DNA in Chlamydomonas had been firmly proven a few years before (Ris and Plaut 1962) and the view was emerging that the photosynthetic machinery is encoded jointly by the nuclear and the chloroplast genome and that chloroplasts contain their own protein synthesizing system with specific 70S ribosomes distinct from the 80S cytosolic ribosomes. The Levine laboratory took advantage of the fact these ribosomes are sensitive to different types of antibiotics to show that several photosynthetic activities depend on chloroplast protein synthesis with the inference that the corresponding proteins must be encoded by the chloroplast genome (Armstrong et al. 1971). These interesting results raised a number of questions regarding the organization, the gene content, and the expression of the chloroplast genome. I was rather fascinated by these questions and decided to switch from the biophysical analysis of photosynthesis to the study of the organization of the chloroplast genome. Paul had expected that as a physicist I would work on the former topic but he graciously accepted to let me work on the chloroplast genome in close collaboration with Steve Surzycki, a post-doctoral fellow from Poland who taught me all the basics of chloroplast molecular biology. Paul was always very supportive, gave critical advice, and encouraged me to pursue my project even if it was not in the mainstream of his research. The atmosphere in the lab was very friendly, relaxed, and stimulating. Each of us could pursue his own ideas. I greatly appreciated the large freedom and support Paul gave me throughout my thesis. These were truly wonderful years which had a lasting effect on my career. Paul respected scientific independence. As an example, he did not wish to be coauthor in the publications resulting from my thesis work (Surzycki and Rochaix 1971; Rochaix 1972).

I had the pleasure to meet Paul on several occasions later. I remember particularly his joyful retirement party in the early 1990s on a large sailing ship in Rhode Island which many of his former post-docs and students attended. While I was on sabbatical at the Carnegie Institution of Stanford in 1998 he invited me with my family at his remote farm close to Monterey, California, where he was studying marine organisms. The access to this farm on a steep bumpy road was quite adventurous. Paul would ride his horses almost every day and my kids had a great time with him during this unforgettable week-end. More recently, I met him when he visited the family of his wife in Switzerland. We had dinner together with a lively discussion on Charles Darwin as this was close to the 200^th^ anniversary of Darwin.

I owe Paul Levine great deal. My stay in his lab as graduate student had a lasting impact on my future research career. This is probably also true for many former members of his laboratory including Nick Gillham, Bob Togasaki, Nam-Hai Chua, Pierre Bennoun, Steve Surzycki, Ursula Goodenough, and others. In the Photosynthesis research community, Paul will be remembered for his pioneering genetic studies of photosynthesis and for laying the ground for a molecular-genetic approach for photosynthesis and chloroplast biogenesis. Paul had wide interests in many fields of biology. Here, I only covered his studies on photosynthesis but he also contributed in the field of complement proteins in immunology during the years he spent in St. Louis.

Finally at an advanced age, he started to write novels. I end my ‘reminiscences’ with a quote from the message Paul Levine sent me barely one month before his death: “At this strange time, each day seems to bring a story often tragic or occasionally a hint of optimism. I'm getting pleasure in the unexpected pleasure that has come from writing crime novels. Two have been finished and perhaps one of them will be accepted by a publisher. Meanwhile, I'm working on number three. My guess is that writing is my way of avoiding thinking about what may become a dystrophic world. Whatever comes I realize that my 95 years have been mostly lucky ones.”
